# Supplementary material for: The genome sequence of a flea beetle, Altica lythri Aubé, 1843
Source: Wellcome Open Res. 2025 Jun 2;10:297. [Version 1] doi: 10.12688/wellcomeopenres.24269.1 (PMC12417985; doi:10.12688/wellcomeopenres.24269.1)
Supplement: Peer review history

# Reviewer report: Reviewer response for version 1

**DOI:** 10.21956/wellcomeopenres.26766.r129640

**Published:** 2025-10-01

**Authors:** Susanne Dobler, Kim Rohlfing

**Affiliations:** 1 Institute of Cell and Systems Biology, Universitat Hamburg, Hamburg, Hamburg, Germany; 2 Institute of Cell and Systems Biology of Animals, Universität Hamburg, Hamburg, Germany

The paper by Paul and colleagues presents a very well curated, high quality genome of a female *Altica lythri* flea beetle. By PacBio and Hi-C sequencing the authors were able to generate a chromosome level assembly (82.3% of sequences in 12 chromosomes) which in addition identifies the X chromosome and mtDNA molecule. Surprisingly as the authors state their GenomeScope and Smudgeplot analysis (which can be found at the ToLQC website) convincingly identify the sequenced individual as triploid. This contrasts with previous publications reporting diploid individuals of the same species and raises interesting questions for our own research.

 Earlier reports (Smith, 1960) already mentioned triploidy and thelytokous parthenogenesis in a North American congeneric species, *Altica lazulina*, but our own extensive work on *A. lythri* suggests that triploidy must be the exception, at least in the populations we monitored. As the authors mention we found three highly divergent mitochondrial haplotypes in *A. lythri*, which coincide with different reproductive modes. Two, which we called HT2 and HT3, show normal sexual reproduction with approximately even ratios of males and females (Jäckel et al., 2013, Rohfing et al., 2024). A male beetle that likely belongs to either of these haplotypes was previously karyotyped (Segarra and Petitpierre, 1982) showing normal metaphase I bivalents of an 11+X+y karyotype. This normal diploid karyotype and reproduction contrasts with the situation observed in HT1/HT1* beetles and based on the mitochondrial DNA data published by Paul et al. the sequenced individual belongs to this mitochondrial type. According to our large scale population screens these individuals are always female and produce exclusively female offspring (Jäckel, 2013; Rohlfing et al., 2023), yet only after copulation with a male of the other haplotypes. This mode of reproduction, called sperm dependent parthenogenesis, could in principle also work for triploid individuals as seen in stick insects (Marescalchi & Scali, 2003).

 Nevertheless, for the well investigated North German populations of *A. lythri* our data rather support at least a majority of diploid individuals. The evidence comes from qPCR based quantifications of the ratio of X chromosomes to autosomes in HT1/1* and HT2 (Rohlfing et al., 2024) and from chromosome counts of unfertilized eggs (unpublished data). Only rare ambiguities in our data can hint to the existence of triploid individuals. This may indicate differences between populations as has been previously observed in other species (Angus, 2025, Nokkala et al., 2008). A screen of more individuals from the same British source population could reveal if Paul et al. sequenced the exceptional individual or if triploidy is actually the rule in this population.

 In conclusion the high quality genome of *A. lythri* is a very helpful resource and indicates exiting avenues for further research into the reproductive biology and population genetics of the species.

 References:

 Angus R.B., 2025, [Reference 1]

 Jäckel R., Mora D., Dobler S., 2013, [Reference 2]

 Marescalchi O., Scali V., 2003, [Reference 3]

 Nokkala S., Maryanska-Nadachowska A., Kuznetsova V.G.,  2008, [Reference 4]

 Rohlfing K., Yue L., Franke S., Zeng C., Podsiadlowski L., Dobler S., [Reference 5]

 Rohlfing K., Grewoldt M., Cordellier M., Dobler S., 2024, [Reference 6]

 Segarra C., Petitpierre E., 1982, Spixiana S7:29-37, https://www.researchgate.net/publication/233777514

 Smith S.G., 1960, Can. J. Gent. Cytol. 2:67-88

Are sufficient details of methods and materials provided to allow replication by others?

Yes

Is the rationale for creating the dataset(s) clearly described?

Yes

Are the datasets clearly presented in a useable and accessible format?

Yes

Are the protocols appropriate and is the work technically sound?

Yes

Reviewer Expertise:

Molecular evolutionary biology, insect-plant interactions

We confirm that we have read this submission and believe that we have an appropriate level of expertise to confirm that it is of an acceptable scientific standard.

## References

[1] : Triploid female Helophorus brevipalpis Bedel, 1881 in Provence, France, with additional data on C-banding in both triploid and diploid material, and discussion of chromosomal variation in H. brevipalpis. *Comparative Cytogenetics* .2025;19: 10.3897/compcytogen.19.162685 125-131 10.3897/compcytogen.19.162685 PMC1231147540747451

[2] : Evidence for selective sweeps by infections: phylogeny of leaf beetles and their reproductive parasites. *Molecular Ecology* .2013;22(16) : 10.1111/mec.12389 4241-4255 10.1111/mec.12389 23927410

[3] : Automictic parthenogenesis in the diploid-triploid stick insectBacillus atticus and its flexibility leading to heterospecific diploid hybrids. *Invertebrate Reproduction & Development* .2003;43(2) : 10.1080/07924259.2003.9652535 163-172 10.1080/07924259.2003.9652535

[4] : First evidence of polyploidy in Psylloidea (Homoptera, Sternorrhyncha): a parthenogenetic population of Cacopsylla myrtilli (W. Wagner, 1947) from northeast Finland is apomictic and triploid. *Genetica* .2008;133(2) : 10.1007/s10709-007-9200-3 201-205 10.1007/s10709-007-9200-3 17851766

[5] : When does the female bias arise? Insights from the sex determination cascade of a flea beetle with a strongly skewed sex ratio. *Functional & Integrative Genomics* .2023;23(2) : 10.1007/s10142-023-01023-1 10.1007/s10142-023-01023-1 PMC1006610837000335

[6] : Evidence for feminized genetic males in a flea beetle using newly identified X‐linked markers. *Ecology and Evolution* .2024;14(8) : 10.1002/ece3.70123 10.1002/ece3.70123 PMC1131810839135725

# Reviewer report: Reviewer response for version 1

**DOI:** 10.21956/wellcomeopenres.26766.r129645

**Published:** 2025-09-08

**Authors:** Jhon Alex Dziechciarz Vidal

**Affiliations:** 1 Universidade Federal de SãoCarlos (UFSCar), São Carlos, Brazil

This data report, entitled “The genome sequence of a flea beetle, *Altica lythri *Aubé, 1843 **”** describes the genome assembly of a female flea beetle, *Altica lythri*. Which is the first genome for this species was deposited at NCBI and provides an important resource for future studies of beetle diversity. The results align with the report’s stated objective: to sequence as many species as possible and make high-quality genomes publicly available.

 The assembly metrics are broadly comparable to those reported for other sequenced beetle genomes. Table 2 summarizes assembly quality; although the scaffold count is relatively high, overall completeness is more than satisfactory. The figures and tables are clearly described and contain the key information needed to assess genome quality and follow the narrative. A few caveats are noted below.

 Using DNA barcoding for specimen identification is essential, as many beetle species are difficult to identify accurately. Sequencing quality is more than satisfactory: the authors targeted long fragment lengths (12–20 kb) with high coverage for PacBio HiFi, together with Illumina Hi-C, both of which are critical for generating a high-quality assembly.

 The genome assembly and curation workflow is clearly described and demonstrates sa trong command of the relevant tools, which are also summarized in Table 4.

**Comments and suggestions**

**Background**

 “ *A. lythri* usually occurs in damp habitats favored by its food plants, including the shores of water bodies, fens, and damp meadows, but it may also frequent dry habitats, including downland and gardens, where it may feed on cultivated plants, including *Fuchsia*.”

 Please cite and link the source for this habitat description so that readers can locate the species in the field.

**Genome Sequence Report**

 The manuscript states that GenomeScope indicates a triploid genome. Please include the GenomeScope plots (in the main text or Supplementary Materials) to substantiate this interpretation. This is noteworthy because prior cytogenetic work on *A. lythri* did not report triploidy (Petitpierre, Segarra, Yadav & Virkki, 1988).

 Figure 5. Please label the chromosomes on the Hi-C contact map to facilitate cross-referencing between chromosome numbers and the contact map.

 Methods

 DNA extraction was performed from the whole organism (“Tissue from the whole organism was homogenized…”). Could whole-body extraction introduce non-target DNA (e.g., endoparasites/parasitoids)? Please comment on contamination controls or screening performed (e.g., dissection strategies, taxonomic/kraken screens, or other contamination checks).

 Table 4: The link “ ftp://ftp.ncbi.nlm.nih.gov/blast/executables/blast+/” is not working.

Are sufficient details of methods and materials provided to allow replication by others?

Yes

Is the rationale for creating the dataset(s) clearly described?

Partly

Are the datasets clearly presented in a useable and accessible format?

Yes

Are the protocols appropriate and is the work technically sound?

Yes

Reviewer Expertise:

Cytogenetics, Animal Genetics, and Genomics.

I confirm that I have read this submission and believe that I have an appropriate level of expertise to confirm that it is of an acceptable scientific standard.

# Reviewer report: Reviewer response for version 1

**DOI:** 10.21956/wellcomeopenres.26766.r125075

**Published:** 2025-08-30

**Authors:** Huai-Jun Xue

**Affiliations:** 1 Nankai University, Tianjin, China

This study is methodologically sound, and the software usage appears to be correct. Based on my experience, the assembled genome size might be slightly larger than expected (I suspect it could be due to high heterozygosity). The GenomeScope analysis also estimated the genome size to be 878M. For reference, one of our published genomes (Altica viridicyanea) is 865M, and the genomes of two other species from the same genus that we are currently assembling are also around 700M.

 The authors addressed “this was a triploid genome”, I guess it is not right. Triploids are rare among insects in nature. In the case of high heterozygosity (for example, 1.5% was estimated in this species), GenomeScope analysis may misjudge diploid as triploid.

Are sufficient details of methods and materials provided to allow replication by others?

Yes

Is the rationale for creating the dataset(s) clearly described?

Yes

Are the datasets clearly presented in a useable and accessible format?

Yes

Are the protocols appropriate and is the work technically sound?

Yes

Reviewer Expertise:

evolution

I confirm that I have read this submission and believe that I have an appropriate level of expertise to confirm that it is of an acceptable scientific standard.
